# Early surgical intervention for patients with possible clinically silent somatotroph adenoma: a case series

**DOI:** 10.1186/s13256-019-1981-3

**Published:** 2019-03-13

**Authors:** Tomohiro Kawaguchi, Yoshikazu Ogawa, Teiji Tominaga

**Affiliations:** 1 Department of Neurosurgery, Tohoku University Graduate School of Medicine, Sendai, Miyagi Japan; 2 Department of Neurosurgery, Kohnan Hospital, 4-20-1 Nagamachi Minami, Taihaku-ku, Sendai, Miyagi 982-8523 Japan

**Keywords:** Acromegaly, Clinically silent, Growth hormone, Somatotroph adenoma, Transsphenoidal surgery

## Abstract

**Introduction:**

Clinically silent somatotroph adenoma is characterized by elevated serum growth hormone but without the clinical symptoms of acromegaly, and it is considered rare. The natural history is not well understood, progress to symptomatic is uncertain, and treatment strategy has not been established.

**Case presentation:**

The first patient was a 48-year-old-Asian woman who presented with serum growth hormone 6.99 ng/ml and insulin-like growth factor 1 of 476 ng/ml, but no characteristic features of acromegaly. Five years after initial diagnosis, she presented with acromegalic facial appearance. Transsphenoidal surgery achieved gross total removal and endocrinological remission. The second patient was a 40-year-old-Asian woman who presented with serum growth hormone 31.14 ng/ml and insulin-like growth factor 1 of 709.6 ng/ml, but no characteristic features of acromegaly. Three years after initial diagnosis, she presented with acromegalic facial appearance. Transsphenoidal surgery achieved gross total removal and endocrinological remission. The third patient was a 64-year-old-Asian woman who presented with serum growth hormone 6.0 ng/ml and insulin-like growth factor 1 of 341 ng/ml, but no characteristic features of acromegaly. Eight months after initial diagnosis, hand enlargement was detected. Transsphenoidal surgery achieved gross total removal and endocrinological remission.

**Conclusion:**

Due to its potential for evolving to symptomatic disease, the risks of surgery and observation for patients with somatotroph adenoma should be carefully compared from the viewpoint of better health outcome.

## Introduction

Somatotroph adenoma is defined as a pituitary neoplasm characterized by unregulated growth hormone (GH) hypersecretion. Typical features such as acral enlargement and coarse facial features identify patients with somatotroph adenomas, leading to a diagnosis of acromegaly. Such patients present with several coexisting clinical conditions, including hypertension, cardiovascular diseases, abnormal glucose tolerance, obstructive sleep apnea, and malignant neoplasms such as colon and thyroid cancer [1–8]. Without treatment, their mortality rate is high compared with the general population [9]. Consequently, early surgical intervention is recommended.

Recently, somatotroph adenomas have been classified according to pathological GH expression and excessive serum GH levels. Adenomas with positive immunohistochemical staining for GH but no elevation of serum GH concentration are considered to be “silent” somatotroph adenoma. Similarly, “clinically silent” adenomas are defined as GH-secreting tumors with elevated serum GH concentration, but without the clinical manifestations of excess GH [10]. This entity of clinically silent somatotroph adenoma is now recognized, but whether clinically silent adenoma will become symptomatic remains uncertain, so no treatment strategy has been established.

We treated three patients with somatotroph adenomas without signs of acromegaly despite elevated serum GH concentration. They developed acromegalic features during follow-up for small sellar tumors and later underwent surgery leading to disappearance of the tumors. We discuss the validity of early surgery for such patients. Informed consent was obtained from all individual participants included in the study.

## Case presentation

### Patient 1

**Fig. 1:**
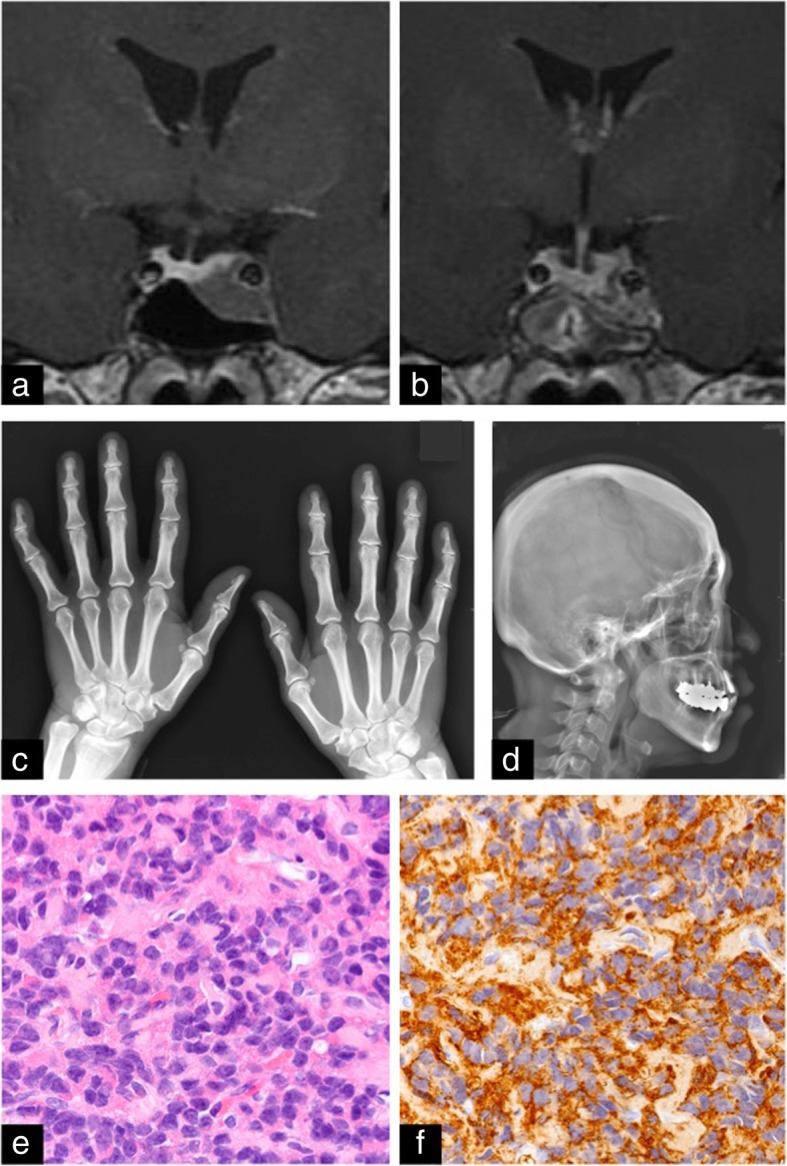
Patient 1. **a** T1-weighted magnetic resonance (MR) image with gadolinium 5 years after initial diagnosis showing a small sellar mass lesion. **b** Postoperative T1-weighted MR image indicating gross total removal of the tumor. **c** Preoperative roentgenogram of the fingers. **d** Preoperative roentgenogram of the face. **e** H&E staining (× 40 magnification) showing sheet-like proliferation of monomorphic round cells. **f** Immunohistochemical staining for growth hormone showing diffuse reactivity (× 40 magnification)

A 48-year-old-Asian woman visited the neurosurgical department of another hospital because of chronic mild headache. Head magnetic resonance (MR) imaging incidentally detected a small mass lesion inside the sella turcica. Endocrinological examination showed high concentrations of serum GH (6.83 ng/ml; normal range, 0–2.47 ng/ml) and insulin-like growth factor 1 (IGF-1) (517 ng/ml; normal range, 82–219 ng/ml). Because she had no neurological deficit or medical history of hypertension and diabetes mellitus, surgery was not proposed at the former hospital, and simple observation was continued. She had no family history of cancer or endocrinological diseases. She occasionally drinks alcohol and has no smoking habit. She worked as a school janitor, and a routine medical checkup showed that her systolic and diastolic blood pressure were around 110 and 70 mmHg, respectively. During the follow-up period, head MR imaging showed no significant change in tumor size, and concentrations of serum GH and IGF-1 were not further increased (6.99 ng/ml and 476 ng/ml, respectively). Five years after the initial diagnosis, baseline blood pressure was elevated to 140/80 mmHg. She had an 8-kg weight gain, and her shoe size was enlarged by 1.5 cm during this period. Finally, she was referred to our department for surgical intervention. Head MR imaging showed that the tumor was slightly enlarged (11 × 16 × 16 mm) and sparsely enhanced with gadolinium (Fig. 1a). On admission, prominent forehead, prominent lower jaw, and bite abnormalities were not observed. A roentgenogram showed cauliflower-like enlargement of the distal phalanx of the fingers (Fig. 1c). The expansion of maxillary or frontal sinus was not particular, but enlargement of the nose and lips was evident (Fig. 1d). Serum concentrations of GH (7.33 ng/ml) and IGF-1 (606 ng/ml) had further increased. A preoperative 75-g oral glucose tolerance test (OGTT) showed no suppression of serum GH concentration. To control excess GH secretion, surgery was proposed. Transsphenoidal surgery achieved gross total removal of the tumor (Fig. 1b). Serum concentrations of GH (1.89 ng/ml) and IGF-1 (422 ng/ml) had rapidly decreased by 1 week after surgery and remained at low levels at 4 months after surgery (GH, 2.91 ng/ml; IGF-1, 339 ng/ml). Postoperative 75-g OGTT showed sufficient suppression of serum GH concentration. She was discharged without neurological deficit. After surgery, the heel pad thickness was decreased by 1 mm, and body weight was decreased by 2.9 kg. The head MR imaging, serum concentration of GH, IGF-1, and 75-g OGTT were followed up for 21 months after surgery, which revealed no evidence of recurrence. Postoperative histological examination of formalin-fixed, paraffin-embedded tumor specimens demonstrated sheet-like proliferation of monomorphic round cells with H&E staining (Fig. 1e), and tumor cells showed diffuse immunoreactivity for GH (Fig. 1f). The histological diagnosis was densely granulated somatotroph adenoma.

### Patient 2

**Fig. 2:**
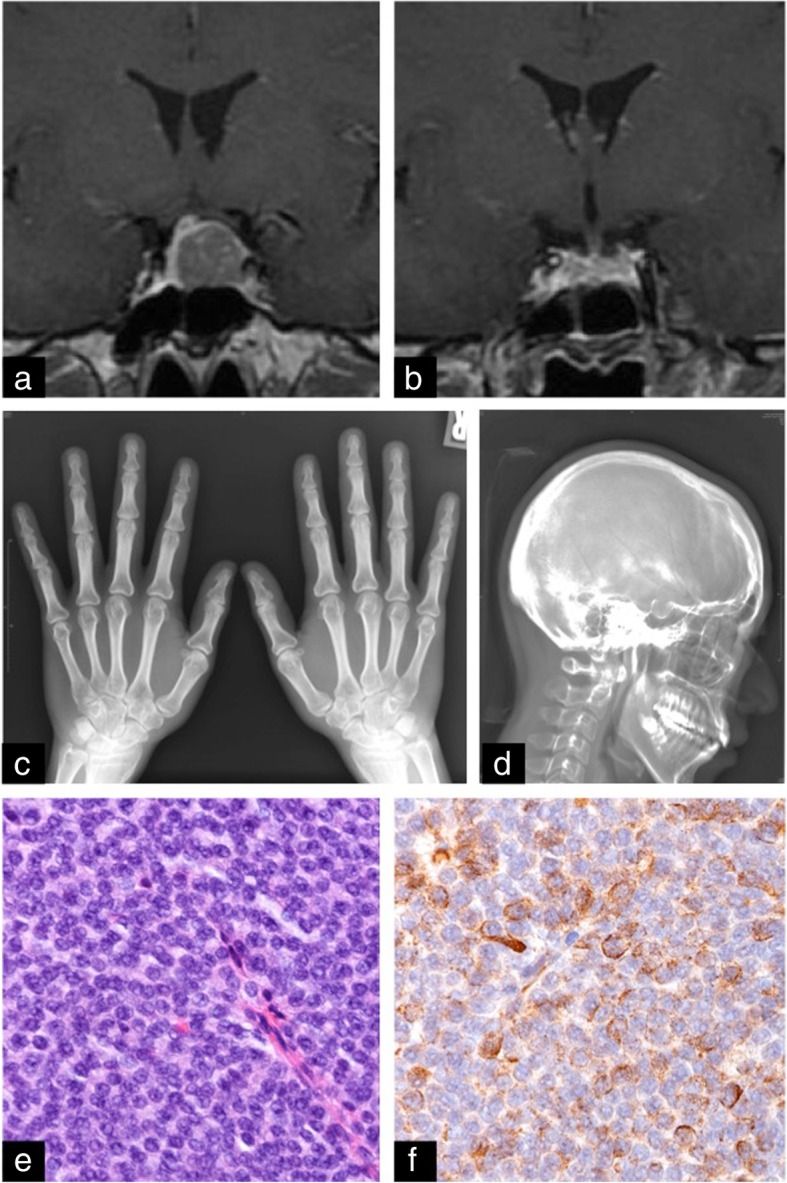
Patient 2. **a** T1-weighted magnetic resonance (MR) image with gadolinium 3 years after initial diagnosis showing a small sellar mass lesion. **b** Postoperative T1-weighted MR image indicating gross total removal of the tumor. **c** Preoperative roentgenogram of the fingers. **d** Preoperative roentgenogram of the face. **e** H&E staining (× 40 magnification) showing sheet-like proliferation of monomorphic round cells. **f** Immunohistochemical staining for growth hormone showing diffuse reactivity (× 40 magnification)

A 40-year-old-Asian woman visited another general neurosurgeon’s clinic because of chronic mild headache. Head MR imaging incidentally detected a small mass lesion inside the sella turcica. She had no family history of cancer or endocrinological diseases. She has no smoking or drinking habit. She worked as an administrative staff member of an office and had no irregular educational and environmental histories. She had no neurological deficit or medical history of hypertension and diabetes mellitus. Endocrinological examination showed high concentrations of serum GH (11.90 ng/ml; normal range, 0.28–1.64 ng/ml). Because she presented no clinical features of acromegaly, the initial diagnostician did not propose surgery, and simple observation was continued. During the follow-up period, head MR imaging showed no significant change in tumor size, and concentration of serum GH was not further increased (10.10 ng/ml). Three years after the initial diagnosis, radiography showed that the tumor had enlarged, and the patient had weight gain and foot size increase. She accepted surgical treatment and was referred to our department. Preoperative MR imaging showed the tumor with suprasellar extension (14 × 19 × 12 mm), and the optic chiasm was slightly compressed upward (Fig. 2a). On admission, prominent forehead, prominent lower jaw, and bite abnormalities were not observed. Roentgenogram showed cauliflower-like enlargement of the distal phalanx of the fingers (Fig. 2c). The expansion of maxillary or frontal sinus was not particular, but enlargement of the nose and lips was evident (Fig. 2d). Serum concentrations of GH (31.14 ng/ml) and IGF-1 (709.6 ng/ml) were further increased. Preoperative 75-g OGTT showed no suppression of serum GH concentration. To control excess GH secretion, surgery was proposed. Transsphenoidal surgery achieved gross total removal of the tumor (Fig. 2b). Serum concentration of GH (0.98 ng/ml) and IGF-1 (407.8 ng/ml) had rapidly decreased by 1 week after surgery and remained at low levels at 4 months after surgery (GH, 0.62 ng/ml; IGF-1, 207.9 ng/ml). Postoperative 75-g OGTT showed sufficient suppression of serum GH concentration. The patient was discharged without neurological deficit. After surgery, the heel pad thickness was decreased by 2 mm, and body weight was decreased by 4.0 kg. The head MR imaging and serum concentration of GH and IGF-1 were followed up for 66 months after surgery, which revealed no evidence of recurrence. Postoperative histological examination of formalin-fixed, paraffin-embedded tumor specimens demonstrated sheet-like proliferation of monomorphic round cells with H&E staining (Fig. 2e), and tumor cells showed patchy immunoreactivity for GH (Fig. 2f). The histological diagnosis was sparsely granulated somatotroph adenoma.

### Patient 3

**Fig. 3:**
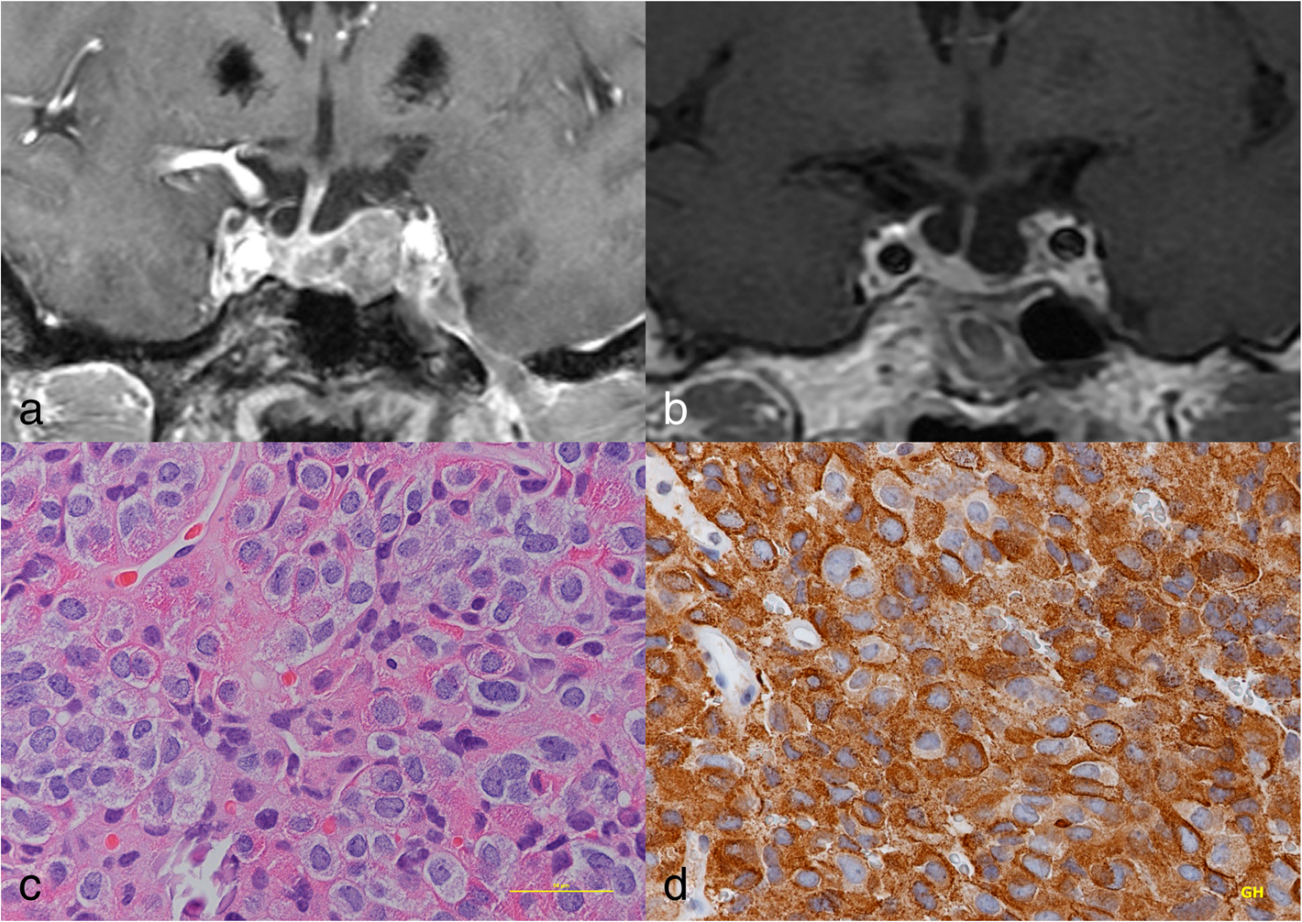
Patient 3. **a** Preoperative T1-weighted magnetic resonance (MR) image with gadolinium showing a sellar mass lesion. **b** Postoperative T1-weighted MR image showing no residual tumor. **c** H&E staining (× 40 magnification) showing sheet-like proliferation of monomorphic round cells. **d** Immunohistochemical staining for growth hormone showing diffuse reactivity (× 40 magnification)

A 64-year-old-Asian woman visited the neurology department of another hospital because of chronic mild headache. Head MR imaging incidentally detected a small mass lesion inside the sella turcica (Fig. 3a), and she was referred to our department. Serum concentrations of GH (6.00 ng/ml) and IGF-1 (341 ng/ml) exceeded the normal ranges, but she had no neurological deficit or medical history of hypertension and diabetes mellitus, and no physical characteristics of acromegaly. She had no family history of cancer or endocrinological diseases. She has no smoking or drinking habit. First, she refused surgery, but 8 months after the initial diagnosis, she accepted intervention. She was a homemaker and had no regular work. She requested the shortest hospital stay and did not agree to the preoperative 75-g OGTT. Preoperative MR imaging showed a slightly enhanced tumor without particular enlargement (11 × 17 × 17 mm). Acromegalic hand enlargement was discovered at the time of surgery. To control excess GH secretion, surgery was proposed. Transsphenoidal surgery achieved gross total removal of the tumor (Fig. 3b). Serum concentrations of GH (0.85 ng/ml) and IGF-1 (104 ng/ml) had rapidly decreased by 1 week after surgery and remained at low levels at 4 months after surgery (GH, 1.76 ng/ml; IGF-1, 174 ng/ml). Postoperative 75-g OGTT showed sufficient suppression of serum GH concentration. She was discharged without neurological deficit. After surgery, her body weight was decreased by 0.5 kg. The head MR imaging, serum concentration of GH, IGF-1, and 75-g OGTT were followed up for 23 months after surgery, which revealed no evidence of recurrence. Postoperative histological examination of formalin-fixed, paraffin-embedded tumor specimens demonstrated sheet-like proliferation of monomorphic round cells with H&E staining (Fig. 3c), and the tumor cells showed diffuse immunoreactivity for GH (Fig. 3d). The histological diagnosis was densely granulated somatotroph adenoma.

## Discussion

Clinically silent somatotroph adenoma was first described in 1985 [11]. Since then, several case reports and case series have been published [10, 12, 13], but this entity is still considered rare. Recently, one-third of somatotroph adenomas were reported as clinically silent, a relatively higher incidence rate than previously believed [10]. However, the natural history of this entity is still unknown. Clinically silent somatotroph adenoma may be biologically active with the potential to become symptomatic [14]. Our series illustrates the natural course of the disease and confirms this potential.

Long-term exposure to excess GH in patients with definitive acromegaly might be associated with the development and progression of comorbidities such as hypertension, diabetes mellitus, cardiovascular diseases, and certain cancers, so early diagnosis and early treatment are recommended to reduce the risk of premature death [15, 16]. However, no treatment strategy has been established for patients with clinically silent somatotroph adenoma, because the risk of clinical symptoms is uncertain. Consequently, surgery for these patients is prophylactic and is only justified if the surgical risk is lower than the risk of simple observation. Morbidity and mortality associated with surgery are decreased if the surgeon has experience of more than 200 procedures [17]. Our institution is a high-volume center for transsphenoidal surgery. The operator (Y.O.) has performed more than 1000 procedures, and the outcomes for the three cases presented here were acceptable. For our patients, medical therapy was a considerable option. Several medicines, such as dopamine agonists or somatostatin analogues, have been advocated, and preoperative medical therapy has been considered useful to obtain enhanced extent of surgical resection and better biochemical remission [18–21]. If the initial diagnostician had chosen medical therapy, our patients possibly could have avoided having acromegalic symptoms. However, the primary mode of therapy for patients with acromegaly is surgery because of better remission rate. Unfortunately, neither surgery nor medical therapy was chosen as an initial treatment for our patients because of patient-related and/or diagnostician-related reasons. To achieve better biochemical remission, surgery should be considered for these patients at the time of initial diagnosis with small sellar mass.

This case series has several limitations. First, the clinical manifestations were initially judged by neurosurgeons. An experienced endocrinologist might have detected subtle features of acromegaly. Second, this report is a three-case series and does not assess the actual rate of evolution to symptomatic disease. Moreover, surgery was performed after symptom onset, so the efficacy of early intervention was not fully addressed. Surgery is prophylactic, so the relative advantages and risks of surgery and simple observation must be considered carefully.

## Conclusion

Although somatotroph adenomas lack the characteristic feature of acromegaly at initial diagnosis, there is a possibility of evolving into symptomatic disease. Further investigation with larger series and longer follow-up is needed.

## Abbreviations

GH: Growth hormone
IGF-1: Insulin-like growth factor 1
MR: Magnetic resonance
OGTT: Oral glucose tolerance test

## References

[1] Boguszewski CL, Boguszewski MC, Kopchick JJ. Growth hormone, insulin-like growth factor system and carcinogenesis. Endokrynol Pol. 2016;67:414–26. 10.5603/EP.a2016.0053 27387246

[2] Bałdys-Waligórska A, Krzentowska A, Gołkowski F, Sokołowski G, Hubalewska-Dydejczyk A. The prevalence of benign and malignant neoplasms in acromegalic patients. Endokrynol Pol. 2010;61:29–34. 20205101

[3] Jenkins PJ, Besser M. Clinical perspective: acromegaly and cancer: a problem. J Clin Endocrinol Metab. 2001;86:2935–41. 10.1210/jcem.86.7.7634 11443146

[4] Wen-Ko C, Szu-Tah C, Feng-Hsuan L, Chen-Nen C, Ming-Hsu W, Jen-Der L. The impact of diabetes mellitus on the survival of patients with acromegaly. Endokrynol Pol. 2016;67:501–6. 10.5603/EP.a2016.0031 26884300

[5] Gasperi M, Martino E, Manetti L, Arosio M, Porretti S, Faglia G, Mariotti S, Colao AM, Lombardi G, Baldelli R, Camanni F, Liuzzi A, Acromegaly Study Group of the Italian Society of Endocrinology. Prevalence of thyroid diseases in patients with acromegaly: results of an Italian multi-center study. J Endocrinol Investig. 2002;25:240–5. 10.1007/BF03343997 11936466

[6] Baroni MG, Giorgino F, Pezzino V, Scaroni C, Avogaro A. Italian Society for the Study of Diabetes (SID)/Italian Endocrinological Society (SIE) guidelines on the treatment of hyperglycemia in Cushing’s syndrome and acromegaly. J Endocrinol Investig. 2016;39:235–55. 10.1007/s40618-015-0404-6 26718207

[7] Terzolo M, Reimondo G, Gasperi M, Cozzi R, Pivonello R, Vitale G, Scillitani A, Attanasio R, cecconi E, Daffara F, Gaja F, Martino E, Lombardi G, Angeli A, Colao A. Colonoscopic screening and follow-up in patients with acromegaly: a multicenter study in Italy. J Clin Endocrinol Metab. 2005;90:84–90. 10.1210/jc.2004-0240 15507515

[8] Wolinski K, Stangierski A, Dyrda K, Nowicka K, Pelka M, Iqbal A, Car A, Lazizi M, Bednarek N, Czamywojtek A, Gurgul E, Ruchala M. Risk of malignant neoplasms in acromegaly: a case-control study. J Endocrinol Investig. 2017;40:319–22. 10.1007/s40618-016-0565-y PMC5331105 27770388

[9] Alexander L, Appleton D, Hall R, Ross WM, Wilkinson R. Epidemiology of acromegaly in the Newcastle region. Clin Endocrinol. 1980;12:71–9. 10.1111/j.1365-2265.1980.tb03135.x 7379316

[10] Wade AN, Baccorn J, Grady MS, Judy KD, O’Rourke DM, Snyder PJ. Clinically silent somatotroph adenomas are common. Eur J Endocrinol. 2011;165:39–44. 10.1530/EJE-11-0216 PMC3118490 21493729

[11] Tourniaire J, Trouillas J, Chalendar D, Bonneton-Emptoz A, Goutelle A, Girod C. Somatotropic adenoma manifested by galactorrhea without acromegaly. J Clin Endocrinol Metab. 1985;61:451–3. 10.1210/jcem-61-3-451 4040530

[12] Trouillas J, Sassolas G, Loras B, Velkeniers B, Raccurt M, Chotard L, Berthezene F, Tourniaire J, Girod C. Somatotropic adenomas without acromegaly. Pathol Res Pract. 1991;187:943–9. 10.1016/s0344-0338(11)81065-4 1792190

[13] Klibanski A, Zervas NT, Kovacs K, Ridgway EC. Clinically silent hypersecretion of growth hormone in patients with pituitary tumors. J Neurosurg. 1987;66:806–11. 10.3171/jns.1987.66.6.0806 3572509

[14] Sakharova AA, Dimaraki EV, Chandler WF, Barkan AL. Clinically silent somatotropinomas may be biochemically active. J Clin Endocrinol Metab. 2005;90:2117–21. 10.1210/jc.2004-0875 15671111

[15] Colao A, Marzullo P, Cuocolo A, Spinelli L, Pivonello R, Bonaduce D, Salvatore M, Lombardi G. Reversal of acromegalic cardiomyopathy in young but not in middle-aged patients after 12 months of treatment with the depot long-acting somatostatin analogue octreotide. Clin Endocrinol. 2003;58:169–76. 10.1046/j.1365-2265.2003.01689.x 12580932

[16] Brue T, Castinetti F. The risks of overlooking the diagnosis of secreting pituitary adenomas. Orphanet J Rare Dis. 2016;11:135. 10.1186/s13023-016-0516-x PMC5052978 27716353

[17] Ciric I, Ragin A, Baumgartner C, Pierce D. Complications of transsphenoidal surgery: results of a nation survey, review of the literature, and personal experience. Neurosurgery. 1997;40:225–36. discussion 236–237 10.1097/00006123-199702000-00001 9007854

[18] Abe T, Lüdecke DK. Effects of preoperative octreotide treatment on different subtypes of 90 GH-secreting pituitary adenomas and outcome in one surgical centre. Eur J Endocrinol. 2001;145:137–45. 10.1530/eje.0.1450137 11454508

[19] Losa M, Mortini P, Urbaz L, Ribotto P, Castrignanó T, Giovanelli M. Presurgical treatment with somatostatin analogs in patients with acromegaly: effects on the remission and complication rates. J Neurosurg. 2006;104:899–906. 10.3171/jns.2006.104.6.899 16776333

[20] Carlsen SM, Lund-Johansen M, Schreiner T, Aanderud S, Johannesen O, Svartberg J, Cooper JG, Hal JK, Fougner SL, Bollerslev J, Preoperative Octreotide Treatment of Acromegaly Study Group. Preoperative octreotide treatment in newly diagnosed acromegalic patients with macroadenomas increases cure short-term postoperative rates: a prospective, randomized trial. J Clin Endocrinol Metab. 2008;93:2984–90. 10.1210/jc.2008-0315 18492760

[21] Mao ZG, Zhu YH, Tang HL, Wang DY, Zhou J, He DS, Lan H, Luo BN, Wang HJ. Preoperative lanreotide treatment in acromegalic patients with macroadenomas increases short-term postoperative cure rates: a prospective, randomised trial. Eur J Endocrinol. 2010;162:661–6. 10.1530/EJE-09-0908 20061334

